# Literature mining of genetic variants for curation: quantifying the importance of supplementary material

**DOI:** 10.1093/database/bau003

**Published:** 2014-02-10

**Authors:** Antonio Jimeno Yepes, Karin Verspoor

**Affiliations:** ^1^National ICT Australia, Victoria Research Laboratory, Melbourne, Australia and ^2^Department of Computing and Information Systems, The University of Melbourne, Melbourne, Australia

## Abstract

A major focus of modern biological research is the understanding of how genomic variation relates to disease. Although there are significant ongoing efforts to capture this understanding in curated resources, much of the information remains locked in unstructured sources, in particular, the scientific literature. Thus, there have been several text mining systems developed to target extraction of mutations and other genetic variation from the literature. We have performed the first study of the use of text mining for the recovery of genetic variants curated directly from the literature. We consider two curated databases, COSMIC (Catalogue Of Somatic Mutations In Cancer) and InSiGHT (International Society for Gastro-intestinal Hereditary Tumours), that contain explicit links to the source literature for each included mutation. Our analysis shows that the recall of the mutations catalogued in the databases using a text mining tool is very low, despite the well-established good performance of the tool and even when the full text of the associated article is available for processing. We demonstrate that this discrepancy can be explained by considering the supplementary material linked to the published articles, not previously considered by text mining tools. Although it is anecdotally known that supplementary material contains ‘all of the information’, and some researchers have speculated about the role of supplementary material (Schenck *et al.* Extraction of genetic mutations associated with cancer from public literature. *J Health Med Inform* 2012;**S2**:2.), our analysis substantiates the significant extent to which this material is critical. Our results highlight the need for literature mining tools to consider not only the narrative content of a publication but also the full set of material related to a publication.

## Introduction

A major thrust of modern biological research is the understanding of how genomic variation relates to disease. This information can be used for disease diagnosis, and increasingly, in the context of personalized medicine, to enable identification of effective disease treatments. There are large-scale efforts to catalogue the results of this research in structured databases, including in the Online Mendelian Inheritance in Man (OMIM) database [1] and the Human Gene Mutation Database (HGMD) [2]. However, much genetic variant information is available only from unstructured sources, including the scientific literature. As such, there have been several systems developed to target extraction of mutations and other genetic variation from the literature [3–9], *inter alia*. Such tools are motivated with claims of their application in the context of database curation [10–13].

The performance of these tools has typically been evaluated *intrinsically*, that is, with respect to a gold standard set of annotations over a corpus of documents. Depending on the precise specification of the task, the gold standard corpus and the tool tested, the performance of these tools has been demonstrated to achieve very high precision and recall [7].

In this work, we instead perform an *extrinsic* evaluation of a mutation extraction tool to test the applicability of text mining, specifically for the curation of mutation databases. This is possible because of the existence of several curated databases that catalogue genetic variants as well as providing links to the source literature, supporting the variation and its disease association. These databases include COSMIC [Catalogue Of Somatic Mutations In Cancer (http://www.sanger.ac.uk/cosmic)] [14] that focuses on somatic mutations, and InSiGHT (International Society for Gastro-intestinal Hereditary Tumours) (http://www.insight-group.org), which targets annotation of the genetic basis of Lynch Syndrome, also known as hereditary nonpolyposis colorectal cancer (HNPCC) [15] within the Human Variome Project (HVP).

Our analysis shows that the recall achieved by the text mining tool in the recovery of mutations catalogued in the databases is very low. Although this effect has been observed previously for protein mutations recorded in the Protein Data Bank (PDB) (http://www.rcsb.org) [16], the work suggested that lack of access to the full-text literature was a major contributor to the problem. In this work, we show that the effect persists even when the full-text article that was indicated to be the direct source of a mutation in a curated resource is available for processing. In one of our evaluations, we find that <3% of curated genetic variants are discovered for the COSMIC database while this value is barely better at just over 8% for the InSiGHT database, even when full text is considered.

We explore several possible explanations for these results, including the inclusion of data from high-throughput experiments, and processing of tables and supplementary material linked to the published articles with the text mining tool. We demonstrate that processing of this additional material enables an increase in recall up to 50%, indicating that most of the curated mutations are not in the abstract or full text of the paper, and that supplementary materials are a critical source of information for curation of genetic variants. Furthermore, our false-negative error analysis shows that the remaining 50% of variants are also available in the supplementary files, but identifying them automatically requires adaptation of current text mining tools to the characteristics of these non-narrative sources of genomic variation data. Our results indicate that to effectively support curation of genetic variants, literature mining tools should consider not only the narrative content of a publication but also the full set of material related to a publication.

## Background

Text mining of mutations in the scientific literature has been addressed by several tools, including MutationMiner [3], MarkerInfoFinder [17], EMU (Extractor of Mutations) [6], MutationFinder [4], tmVar [9] and SETH [18]. A thorough review can be found in [7]. These tools have been shown to achieve a performance over 90% F-measure, and in some cases perfect Precision/Recall, on intrinsic evaluations. There are also several corpora that are publicly available to support intrinsic evaluation of mutation extraction tools [4, 6, 17, 19–21].

There has been some work in assessing the ability of mutation extraction tools to recover the information in a curated mutation resource. Krallinger *et al.* [5] provided a targeted study of mutations occurring in the protein kinase domain (using a system that in later work appears to be referred to as SNP2L [22]). Their strategy was to identify a corpus of kinase domain articles, identify all the mutations mentioned in those articles and then assess overlap of those mutations with curated resources. Using both abstracts and full texts, they showed that their approach was able to recover ∼50% of the mutations in two databases, KinMutBase [23] and the Swissprot Variant database [24], but only 20% of the mutations in SAAPdb [25] and a small fraction (6%) of the mutations in the COSMIC database [14], at the time of their study in March 2009.

Caporaso *et al.* [16] explored the ability of the MutationFinder tool to recover protein mutations annotated in PDB protein records. They considered 70 PDB records with 13 mutations, and the corresponding primary citations of those records, finding that <10% of these mutations could be recovered from abstracts, while >70% were able to be recovered from the full-text articles. The authors concluded, as follows from the results of Krallinger’s work and as we will also show, that a system’s performance on gold standard data is not necessarily indicative of its applicability to large-scale biocuration tasks. This finding has been echoed in similar contexts [19, 26], in which protein residue information extracted from full-text documents lacked coverage compared with existing PDB entries.

Rance *et al.* [13] applied the EMU tool to identify genetic variants associated with drugs. Their study was limited to 104 abstracts in the PharmGKB “VIP” (very important pharmacogenes) dataset; this is a set of manually curated articles in PharmGKB [27]. For the 33 abstracts with corresponding full-text articles, they were able to increase overall recall of gene–drug relations from 33 to 48% by analysing the full text. Hakenberg *et al.* [8] mine PubMed for associating genetic variants with drug, diseases and adverse reactions, and evaluate coverage of these associations, also using PharmGKB. For gene-variant relations, they found that their SNPshot method recovered 96.5% of the PharmGKB gene-variant annotations, after processing nearly 180 000 PubMed abstracts, though for gene-RefSNP annotations the coverage dropped to 65.4%. These results are significantly higher than the others we have reported on, perhaps because by processing significantly more literature, they are increasing the chances of finding any individual gene-variant association. The detailed analysis of 40 “VIP” genes showed lower recall (73.4%) for gene-variant relations and a precision of 58.8%. As PharmGKB is focused specifically on curation of gene–drug relationships, it is also possible that evaluation against this resource is not representative of the general problem of exhaustive annotation of genetic variants. This assertion is supported by the fact that the 96.5% coverage is accounted for by only 505 individual gene-variant associations (where we find several orders of magnitude more variants in the COSMIC database, Table 1).

**Table 1.** COSMIC and InSiGHT data set statistics. Each row reflects figures for cited articles (PMIDs) in the reference database

Set	PMIDs	Mut Art	Mut Cnt	Avg Mut	SD
COSMIC (reference)	9950	7898	198 864	25.18	521.18
InSiGHT (reference)	809	809	7022	8.68	18.55

Mut Art = number of articles associated to at least one mutation; Mut Cnt = the number of mutations associated with those articles; Avg Mut = the average number of mutations per article; SD = standard deviation of Mut Cnt.

Taken together, these prior results not only indicate that processing full text is essential for supporting curation of genetic variants, both in proteins and DNA, but also raise doubts about the role of text mining tools in the context of real-world curation tasks. In some ways, the real-world scenarios tested have been difficult — SNP2L and SNPshot considered a broad set of literature, not specifically tied to a database — whereas in other ways, these evaluations have been too limited to draw reasonably generalizable conclusions, focusing on a small number of papers, or a narrow biological context. Our investigation addresses both of these issues by expanding the scope of analysis to a larger set of genetic variants, while also focusing on the more straightforward task of reproducing manual annotation of specific articles that have been explicitly indicated to be the source of a given curated mutation. This allows us to test directly how well text mining tools can approximate the performance of human curators who work to extract specific gene-variant information from individual articles.

## Methods

To investigate the ability of mutation extraction tools to recover mutation information curated directly from the literature, we required a curated database that includes mutations plus specific links to the literature (with PubMed identifiers [PMIDs] included for each mutation). We selected the COSMIC and InSiGHT databases for our investigation. These databases are used as reference sets; the information extracted from the corresponding scientific literature is compared directly with the information curated from those articles in the databases. We normalize mutation mentions to Human Genome Variation Society (HGVS) format [28].

### The mutation databases

COSMIC [26] (COSMIC site: http://www.sanger.ac.uk/cosmic) contains comprehensive curated information on somatic mutations in human cancer. We used version v62 (from 29 November 2012) available from COSMIC’s FTP site (COSMIC’s FTP site: ftp://ftp.sanger.ac.uk/pub/CGP/cosmic), including mutation information curated from 9950 unique PubMed articles, as well as Cancer Genome Project (CGP) (Cancer Genome Project site: http://www.sanger.ac.uk/genetics/CGP) studies and international system screens [e.g. International Cancer Genome Consortium (ICGC (International Cancer Genome Consortium site: http://dcc.icgc.org/web)]. We identified 7898 publications associated with mutation information in this resource. cDNA and protein mutation information is already available in HGVS format. Genes are referenced by name and by HGNC (HUGO Gene Nomenclature Committee) identifier.

InSiGHT maintains a database of genetic variants for both Lynch Syndrome and Familial Adenomatous Polyposis. The current database has only curated mutations for four genes: MLH1, MSH2, MSH6 and PMS2. The original database was established in the 1990s with mutations reported by individual laboratories. Reports manually extracted from published literature comprise the majority of entries in the InSiGHT database (∼75%, according to the database curator), with the balance direct submissions from clinics.

We accessed the InSiGHT database on 2 January 2013 to establish our data set. The data include variants with curated associations linked to 809 PubMed citations. The database contains information about the variants in the fields *Variant/DNA* and *Variant/Protein*. The amino acids in protein variants have been normalized to single letter amino acid abbreviation form.

There are 41 articles that have been curated both in COSMIC and InSiGHT databases. Unfortunately, none of the mutations in the overlapping articles has been curated by both databases because COSMIC is focused on somatic mutations, whereas InSiGHT is focused on germline mutations in only four genes.

### Corpus collection

An abstract for each PMID was retrieved from Medical Literature Analysis and Retrieval System Online (MEDLINE) using National Center for Biotechnology Information (NCBI)’s E-Utils (NCBI Entrez Programming Utilities Help: http://www.ncbi.nlm.nih.gov/books/NBK25500). Abstracts are downloaded in XML format, and XML-escaped characters are converted to their text characters (e.g. A-&gt;T becomes A–*>*T). In the case of the COSMIC database, 17 articles do not seem to be available when querying PubMed.

A small portion of PubMed is available as full-text articles through the Open Access collection in PubMed Central (PMC-OA). From the 9950 PMIDs available from the COSMIC set, only 563 were available from PMC-OA (5.7%). From the 809 citations for InSiGHT, only 13 were available through the full-text PMC-OA (1.6%).

We attempted to extend this set by retrieving HTML of full-text articles from publisher websites, filtering out those that were found to contain a title and an abstract, but no body. The HTML was converted into text and processed to remove irrelevant information, such as references. Combined with the PMC-OA set, we could recover 2395 full-text articles for the COSMIC database and 165 for the InSiGHT database.

### Mutation identification in text

We selected the EMU tool [6] to process the corpora. EMU was designed to capture a broader range of mutations than other tools, and hence is a better fit for the variation we might expect to find. It identifies protein and DNA point mutations, dbSNP identifiers [29] (RSIDs) and DNA insertions and deletions. In addition, it links the mutations to the proteins and genes that appear in text and performs verifications using existing databases to increase the precision of the annotations. EMU has been shown to have a performance of 0.92 F-score on an intrinsic evaluation, i.e. it has high recall and high precision. EMU version 1.0.19 was used. This version has an available option to process the text either one sentence at a time or across the whole text, which impacts the scope at which links between mutations and genes are identified (i.e. only within a sentence or in the whole text). We used the option to process the whole text because our aim is to maximize the coverage, and that option is less restrictive. Our evaluation in full-text extraction did not show a significant difference in coverage while showing a smaller set of mutation + gene pairs, meaning that gene and mutation co-occur in the same sentence. In addition, EMU can increase the precision of the predicted mutations by performing sequential checking. We have not used this option, again to maximize the coverage of the extracted variants with respect to the reference set. Table 2 shows the number of articles with at least one identified mutation and the number of mentions per category. We ignored the genome category because genome variants do not appear in COSMIC or InSiGHT.

**Table 2.** Counts of mutations identified by EMU, by corpus and by mutation type

Set	COSMIC		InSiGHT	
	Abstracts	Full text	Abstracts	Full text
Papers with mutation mentions	2486	2395	235	165
DNA	139	623	165	97
Genome	21	10	3	5
Protein	3266	18 015	283	1071
Protein; DNA	786	3229	137	269
Protein; DNA; RNA	118	517	32	132
RSID	55	275	14	92
All	4267	22 575	602	1593
Average	1.76	9.43	2.67	9.65
SD	1.44	16.94	2.96	16.87
Mutations with no gene	542	48	110	1
Papers w/HGVS mutation + gene	2373	2251	195	150
HGVS mutation + gene count	8960	57 369	1649	12 908
Average	3.78	71.66	8.45	86.05
SD	4.61	148.36	17.11	225.52

The table shows the statistics after normalizing the mutations to HGVS and assigning one related gene per mutation. (insert this as a footnote for this table)

We normalized the mutation mentions identified by EMU to the HGVS format to be comparable with the information in the COSMIC and InSiGHT databases. Missense mutations, mutations in the DNA that result in a protein change, identified by EMU as *PROTEIN*, are normalized to amino acid (wild-type), position, amino acid (mutated), using single-letter amino acid abbreviations. Thus, a mutation identified by EMU with wild-type amino acid *Ala*, position *140* and mutated amino acid *Thr* is converted to *A140T*. DNA mutations identified by EMU are normalized to the format ‘c.[position][wild type nucleotide] *> *[mutated nucleotide]’. In the case of insertion and deletions, given position ranges, hyphens are replaced by the underscore character (e.g. *c.597-598delGA* to *c.597_598delGA*). When EMU identifies a dbSNP identifier, the dbSNP API is queried to obtain further details about the mutations, identifying all available candidates for DNA and protein mutations associated with each ID.

There were mentions in which the position of the DNA or protein substitution mutation was provided as exon/intron number or a codon position. The codon positions were converted to the three-candidate nucleotide positions. Exon and intron mentions were removed because no precise position could be derived.

EMU identifies gene mentions based on matching a dictionary of human gene names compiled from the Human Genome Organization (HUGO) and NCBI’s gene database. From this dictionary, gene names identical to codon names were removed and ‘P53’, absent in both source dictionaries, was added. InSiGHT curated genes are easy to map, as only 4 genes are included. The COSMIC database contains the gene name and, in most cases, a reference to HGNC. We mapped the gene mentions identified by EMU to HGNC identifiers. The gene name is normalized to the NCBI Gene database, and then mapped to the HGNC identifier.

Table 2 shows the statistics on the number of articles for which we could normalize the extracted mutations to HGVS format. It also shows the number of unique normalized mutations (HGVS mutation) and gene pair. Because the assignment of genes to mutations is done based on co-occurrences, sometimes a mutation is assigned to several genes.

### Collection of additional material linked to the articles

In addition to narrative text, we have analysed further content linked to the papers, which includes the tables and supplementary material. We collected articles from COSMIC and InSiGHT that are available in the open access subset of PubMed Central (PMC-OA), as it already contains the tables in the XML of the article and there are explicit links to the supplementary material. The open access literature has been shown to be representative of the broader biomedical literature in terms of textual characteristics [30]; hence, we expect that our analysis would generalize across PubMed. For the set of 13 articles in the InSiGHT database that could be found in PMC-OA, InSiGHT contains 252 mutation triples. COSMIC associates 33 814 mutation triples to the 563 articles in PMC-OA.

We extracted the tables and table captions from the full-text PMC-OA articles and processed them with EMU. The COSMIC database references 394 PMC-OA articles with tables; 197 of these were identified as having mutations in the tables. From the InSiGHT database, there are only eight articles with tables, of which four contain mutations. In these articles, no mutations were found in the abstract or full text.

Supplementary material was also identified from links within the PMC articles and downloaded. The InSiGHT set contains a limited number of supplementary material files (in 1/13 articles), whereas COSMIC has a larger number linked to the papers (in 138/563 articles). In contrast to PMC-OA articles, available in XML following a consistent DTD, supplementary material appears in a variety of file formats. The most frequent types of supplementary material in this corpus are in order of frequency: MS Word documents, MS Excel spreadsheet, PDF documents, TIFF images and MS Powerpoint documents. Text from the supplementary material was extracted with Apache Tika 1.3 (http://tika.apache.org/1.3) and then processed with EMU. No image processing was performed.

During the extraction of tables and supplementary material, we realized that some PubMed Central articles do not contain the full text in XML format but a link to a PDF version of it. From the InSiGHT collection, four papers out of the 13 contained only the abstract with a link to the full text in PDF format. In the COSMIC collection, the proportion is 76 papers out of 563. The PDF version for these papers has been downloaded from the European PubMed Central mirror (http://europepmc.org), which offers a straightforward link to download the PDF files. The documents were converted into text using Apache Tika 1.3.

## Results

We compare the curated variants in the COSMIC and InSiGHT databases with the information extracted from the literature using EMU. Table 1 shows the distribution of mutations associated to articles in each database. One notable statistic in the COSMIC database is the large number of mutations associated with each PMID article on average (Avg Mut = 25.18) and the large variation (Std Dev = 521.18). In the InSiGHT database, the average number of mutations per article is much lower than in the COSMIC database. This might be explained partially by the focus of the InSiGHT database, i.e. the limited number of genes related to Lynch syndrome.

Despite recovering most of the abstracts from MEDLINE, a limited proportion of those abstracts contain any mutation mentions when processed with the automatic mutation extraction tool [2373/9936 (23.88%) in COSMIC and 195/809 (24.10%) in InSiGHT]. As mutation extraction algorithms have high performance, we can safely assume that this information was not available in the abstracts. To verify this assumption, we manually analysed 100 randomly selected abstracts for each of the two databases. If at least one mutation mention was found that provided enough information to be converted into HGVS format, the abstract was counted as a positive example. In this sample, we found that only 22% of abstracts for the COSMIC database and 23% of abstracts for the InSiGHT database contained at least one mutation mention. As this result is in agreement with the findings of the automatic processing done with EMU, our assumption is supported.

Only a small portion of the articles in each database could be recovered as full text, but we find that a larger proportion of full-text articles contain mutation mentions (2251/2394 in COSMIC and 150/228 in InSiGHT). The proportion of available full-text articles with at least one mutation is higher than in the case of the abstracts.

Table 3 examines the recovery of information extracted from the abstracts (*Abs*), the full text (*FT*) and their combination (*Abs + FT*), for each reference database. The *Cmn Art* (Common Articles) column shows the number of articles associated with mutations in the reference database that also had automatically extracted mutations, for each subcorpus (Abs, FT or Abs + FT). In Table 1, we see that only 7898 articles (*Mut Art*) out of 9950 referenced in the COSMIC database are linked to DNA or protein mutations; the gap results from some articles referencing non-coding variants not formally recorded as mutations in COSMIC (personal communication, COSMIC database curators) and does not affect the calculation of mutation recall. This gap also explains why the *Cmn Art* set for COSMIC in Table 3 is not the full set of articles for which EMU extracted mutations (2200/2373 abstracts and 2071/2251 full-text articles), as EMU may find mentions of these unrecorded variants. The problem does not affect InSiGHT.

**Table 3.** Recall of COSMIC and InSiGHT curated mutations, evaluated over the full reference database (Recall), articles common to each subcorpus and the reference database (Cmn Art) (Recall Common), and considering relaxation of gene match for each case (NG = no gene; Recall NG/Recall CmnNG)

Set	Cmn art	Match mutation	Recall	Recall NG	Mutations common	Recall common	Recall CmnNG
COSMIC Abs	2200	1884	0.0095	0.0122	12,940	0.1456	0.1875
COSMIC FT	2071	3656	0.0184	0.0215	104,756	0.0349	0.0408
COSMIC Abs + FT	3738	4754	0.0239	0.0289	114,279	0.0416	0.0503
InSiGHT Abs	195	230	0.0328	0.0450	1233	0.1865	0.2562
InSiGHT FT	150	404	0.0575	0.0612	1626	0.2484	0.2644
InSiGHT Abs + FT	295	588	0.0837	0.0961	2657	0.2213	0.2540

We also find that the number of identified mutations by EMU is larger than the mutations matched to curated variants, as previously observed by Schenck et al. [31] when using MutationFinder to annotate COSMIC related papers. There are several reasons for mutations in the articles not being curated in the databases. One reason is that there are mutations in the papers that are not of interest to the databases, e.g. the scope of COSMIC is somatic mutations only while InSiGHT focuses on germline mutations in only four genes. Another issue is that some of the reported mutations have been found not to be relevant to the disease under study, e.g. the mutation is not related to the disease, and such negative results are not curated. Further work is required to catalogue or filter the mutations before providing them to a database curator.

We calculate the recall obtained when matching the gene and either the DNA or protein mutation for each record in the database (*Recall*) as well as when the gene is ignored (*Recall NG (No Gene)*), to allow for incorrect normalization of the gene name. We also calculate recall only with respect to mutations directly associated with the common articles (*Cmn Art*) set (*Recall Common* and *Recall CmnNG*) to focus the assessment on those articles (a) that the system had access to, and (b) for which EMU had at least one positive extraction. We find that the recall is low, especially for COSMIC. We recover only 2% of the full set of curated mutations in COSMIC and not even 9% for InSiGHT. Recall over full-text articles is higher than for abstracts, even though the number of articles is smaller. The combination of abstract and full text has a much higher coverage on both sets, though still barely exceeds 5% for COSMIC under the most generous evaluation conditions and 25% for InSiGHT.

We have performed two further analyses to better understand the results. In the first one, we compared the information extracted from the abstract citations with the information extracted from the full-text articles. In the second analysis, we explored the assumption that citations in the COSMIC database reporting a larger number of mutations might have putative assertions that are not reported in the text of the article but rather in the supplementary material, which might explain the low recall of our initial experiments.

### Abstracts versus full text

In this experiment, we consider only articles (*Art Set*) for which at least one mutation is found in the reference database, in the full-text set and in the abstract set (i.e. the three-way intersection). In Table 4, we show that more mutations are extracted from full text in both databases. We have checked whether there is information extracted from the abstracts that is missing from the full text, looking at the precision of the information extracted in the abstract, using the full text as reference. Precision is <96% for COSMIC and <92% for InSiGHT. This means that most, but not all, of the information extracted from the abstracts can be found in the corresponding full text. A closer look shows that the difference is due to conversion issues for the full-text articles (i.e. in article PMID: 9927033, *C676T → Arg226Stop* in the abstract versus the *C676T → Arg226Stop* in the HTML version of the full text, which was converted to the non-standard *C676T_Arg226Stop* during pre-processing) that resulted in EMU missing some mention in the full text.

**Table 4.** COSMIC and InSiGHT results on common articles

Set	Art set	Match mutation	Recall common	Recall commonNG
COSMIC Abs	822	806	0.2272	0.2740
COSMIC FT	822	1310	0.3692	0.4247
InSiGHT Abs	50	50	0.2475	0.3713
InSiGHT FT	50	90	0.4455	0.4950

### High-throughput set in COSMIC

The distribution of mutations in Table 1 shows that there are curated articles with a large number of mutations. We hypothesized that these are publications that make use of high-throughput methods for genetic variant analysis, and that there would be important differences in the performance of the automatic mutation extraction on these types of articles.

We divided COSMIC into two groups, high-throughput (HT) studies or not (NHT), using the MeSH headings available in MEDLINE for each article. Articles were labeled as HT studies if they contain any of the MeSH headings in Table 5 or any of their descendants in the MeSH hierarchy.

**Table 5.** MeSH headings denoting high-throughput papers

MeSH heading	MeSH tree code
Computational biology	H01.158.273.180
Genetic techniques	E05.393
Genome	G05.360.340
Molecular sequence data	L01.453.245.667
Proteome	D12.776.817
Proteomics	H01.181.122.738

We evaluated this labeling procedure on the NHGRI (National Human Genome Research Institute) catalogue of Genome-Wide Association Studies (http://www.genome.gov/GWAStudies), identifying 1466 PMIDs for studies indexed there. Using our MeSH heading-based strategy, 1283 of these articles were labeled as HT, representing 87.34% recall of the classification procedure on this set. Error analysis showed that not all of the GWAS PMIDs had been indexed with MeSH key terms, which is often the case for recently indexed publications. Redoing the calculation without considering these, we labeled 1334 articles as HT, with a recall on this set of 96.32%. We performed a second evaluation of the top 50 articles in the catalogue, ranked by the number of genes in the study (provided as a private communication by National Human Genome Research Institute), and found that 49 (98%) had one of the MeSH headings we consider. The missed article did not contain a MeSH heading that would be related to this topic. These results show that the MeSH headings of articles indexed in PubMed are good indicators for identifying high-throughput studies.

Looking at the indexed PMIDs missed by this procedure, we find that most of them are older articles. For instance, the MeSH headings *Genome-Wide Association Study* and *Genome Association Studies* have been available only since 2009 and 2010, respectively. Many papers are older than these dates. For instance, the PMID 17053108 is from 2006 and does not contain a MeSH heading relevant to the high-throughput topic. This is because when new terms are added to MeSH, they are not applied to articles already indexed.

We labeled the citations in COSMIC using the procedure based on MeSH headings. Table 6 provides the distribution of mutation associations after the labeling. The HT articles (79% of referenced articles) contain <94% of the mutations in the COSMIC database (*Mut Recall*) and account for the high average number of mutations in COSMIC. In Table 7, we see the results of EMU over the COSMIC HT and COSMIC NHT subsets, respectively. The NHT group shows a much larger recall compared with the HT group, supporting the hypothesis that the HT articles pose a particular challenge for the automated extraction methods.

**Table 6.** COSMIC high-throughput (HT)/non-high throughput (NHT) subset statistics

Group	PMIDs	Count	Average mutation	SD	Mutation recall
COSMIC	7898	198 864	25.18	521.27	100.00%
COSMIC-HT	6266	187 367	29.90	584.82	94.22%
COSMIC-NHT	1632	11 497	7.04	38.05	5.78%

**Table 7.** COSMIC High-Throughput (HT)/Non-High Throughput (NHT) subsets, mutation extraction results

Set	Cmn art	Match mutation	Recall	Recall NG	Recall common	Recall CmnNG
HT abstract	1650	1357	0.0072	0.0096	0.1209	0.1608
HT full text	1545	2719	0.0145	0.0172	0.0270	0.0319
HT Abs + FT	2608	3501	0.0187	0.0231	0.0320	0.0395
NHT abstract	550	530	0.0461	0.0543	0.3055	0.3597
NHT full text	526	937	0.0815	0.0915	0.2350	0.2639
NHT Abs + FT	841	1259	0.1090	0.1243	0.2538	0.2895

To assess possible bias in the analysis described in Section 3.4 across these two subsets, we considered the proportion of each corpus available through PMC-OA. For the full COSMIC corpus, 6% (479 articles) are available in PMC-OA, with slightly more of the COSMIC-NHT subset being available in the open access collection (136 articles, 8.3% of the COSMIC-NHT subset) than COSMIC-HT (343 articles, 5.5% of the COSMIC-HT subset). The PMC-OA subcorpus we considered is, therefore, reasonably balanced across the two subsets; the analysis of the collected PMC-OA articles should be representative of both groups.

As a complementary study, we analysed the mutation extraction performance at several mutation frequency thresholds. As shown in Table 8, we find that most of the articles (96%) have <30 curated mutations in COSMIC; these articles account for <20% of the curated mutations. Only 4% of the articles contain 80% of the mutations.

**Table 8.** Descriptive statistics of mutations in COSMIC, grouped by the number of mutations per curated article

Group	PMIDs	Count	Average mutation	SD	Mutation recall
COSMIC	7898	198 864	25.18	521.27	100.00%
COSMIC ≤ 10	6549	20 491	3.13	2.50	10.30%
COSMIC ≤ 20	7339	31 814	4.33	4.30	16.00%
COSMIC ≤ 30	7589	38 015	5.01	5.61	19.12%
COSMIC *> *30	309	160 849	520.55	2590.32	80.88%

Table 9 shows the mutation extraction results at each threshold. We find that with lower threshold values, the recall is higher, which seems to indicate that it is more likely that the mutations are identified in text. In addition, we find that the mutations found in the abstracts and full text seem to be complementary. Considering the set of articles with >30 mutations, the recall drops considerably compared with the other thresholds.

**Table 9.** COSMIC mutation extraction results at several frequency thresholds

Set	Cmn art	Match mutation	Recall	Recall NG	Recall common	Recall CmnNG
C ≤ 10 Abstract	2024	1700	0.0830	0.1054	0.3460	0.4394
C ≤ 10 Full text	1664	2172	0.1060	0.1230	0.4218	0.4894
C ≤ 10 Abs + FT	2941	3719	0.1551	0.1880	0.3838	0.4652
C ≤ 20 Abstract	2144	1832	0.0576	0.0743	0.2780	0.3586
C ≤ 20 Full text	1885	3449	0.0916	0.1084	0.3510	0.4153
C ≤ 20 Abs + FT	3233	3988	0.1253	0.1537	0.3205	0.3930
C ≤ 30 Abstract	2171	1858	0.0489	0.0631	0.2568	0.3317
C ≤ 30 Full text	1969	3299	0.0868	0.1014	0.3179	0.3712
C ≤ 30 Abs + FT	3330	4381	0.1152	0.1397	0.2955	0.3583
C *> *30 Abstract	29	29	0.0002	0.0002	0.0051	0.0051
C *> *30 Full text	102	357	0.0022	0.0026	0.0038	0.0045
C *> *30 Abs + FT	119	373	0.0023	0.0027	0.0038	0.0044

### Analysis of additional material

Our results demonstrate that the vast majority of mutations curated from the literature cannot be automatically identified in the abstract or in the full text of the curated articles. This suggests that these mutations are not mentioned in the narrative content of the articles but must appear in parts of the article that are not being processed by the text mining methods. We know, for instance, that important content appears not only in the body of an article but also in tables [32]. Our analysis of the high-throughput articles also led to consideration of another important source of variants, information external to, but associated with, the main article, such as supplementary material. It is particularly likely that a high-throughput study would exclude a large number of results from the narrative content. We, therefore, performed an experiment to verify this hypothesis.

**Figure 1.**
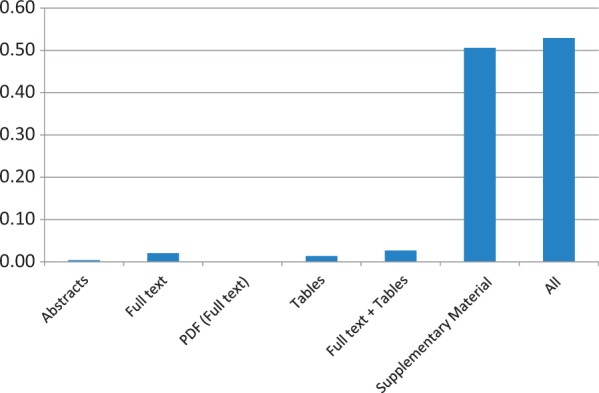
COSMIC data set recall results of applying EMU to different sources and their aggregation (All) on the PMC set. Matching of the triple PMID, gene and mutation is required to obtain a match.

**Figure 2.**
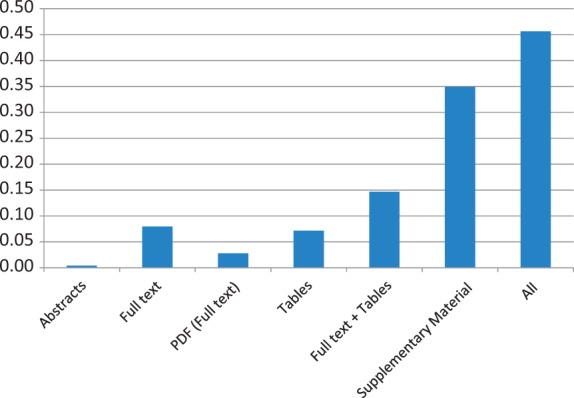
InSiGHT data set recall results of applying EMU to different sources and their aggregation (All) on the PMC set. Matching of the triple PMID, gene and mutation is required to obtain a match.

As above, coverage of mutation extraction was evaluated by matching the triples (PMID, gene, mutation) extracted by EMU with the curated mutations in COSMIC and InSiGHT. Table 10 and Figures 1 and 2 summarize the mutations matched and the recall, by database and article source. The full-text coverage does not get much higher using the PDF representation of the full-text articles where full-text XML was not available from PMC. Tables do contain some mutations, although they only add a limited amount of information. Combining the information from the full text and the tables (*Full text + Tables*) shows that these sources are mostly complementary, indicating that mutations in tables are not repeated in the full text. Finally, we find that supplementary material has the largest coverage, exceeding any other mutation source considered by far. The combination of all the sources reaches >45% in the case of the InSIGHT database, and is >52% for the COSMIC database. These results confirm our suspicion that most of the mutations being curated in the studied databases, are not in the narrative text of the article but appear in supplementary material and, in lower quantity, in tables.

**Table 10.** Mutation extraction results from several mutation sources for the PMC articles

	COSMIC		InSiGHT	
Set	Matched	Recall	Matched	Recall
Abstracts	140	0.0041	1	0.0040
Full text	694	0.0205	20	0.0794
PDF (full text)	23	0.0007	7	0.0278
Tables	466	0.0138	18	0.0714
Full text + tables	906	0.0268	37	0.1468
Supplementary Material	17 015	0.5059	88	0.3492
All	17 896	0.5292	115	0.4563

Results are compared with 252 mutations linked to PMC articles in InSiGHT and 33 814 mutations in COSMIC. PDF refers to a full-text publication only available as PDF.

Despite the dramatic increase in recall by processing the supplementary material, it is still limited to ∼50%. To understand why there remains a substantial gap in recall, we performed an error analysis of a selection of false negatives, i.e. mutations missed by the text mining. Considering the false negatives from the COSMIC database, we find that most of the mutations belong to two articles (PMID:21720365 with 7878 mutations and PMID:22622578 with 1550 mutations). Because a random selection of mutations from COSMIC would likely return mutations only for these two articles, we have done three separate random selections: for each of these two articles, a selection of 10 mutations from the set of false negatives, as well as 85 mutations from the set of false negatives remaining after removing these two articles.

The analysis of the false negatives from PMID:21720365 shows that the mutations can be found in a supplementary plain text file. However, the format of this file is not standardized and mutations are spread across several fields within the file, which explains why EMU did not return any of these mutations. On the other hand, the false negatives from PMID:22622578 are in an MS Excel supplementary file. Interestingly, the mutations do appear in HGVS format, but the position numbering differs from the curated mutations, indicating that curators adjusted the numbering to a (different) reference sequence (genome build). This was the only paper in our analysis that was found to use a different reference sequence numbering for the mutations, preventing a direct match to the information in the database.

Analysing the randomly selected false negatives from the rest of the articles, we also find that the missing mutations do appear in the supplementary material and, less frequently, in the tables linked to the articles or in the full text. In many cases, we find that mutations in MS Excel files were not in HGVS format and the position and other details about the mutation were spread among several fields in these files. In some other articles, e.g. PMID:21750719, PMID:22237025 and PMID:22675565, the chomosome position of the mutation was provided, requiring further processing to determine a gene-based position.

Although less frequent, EMU did not seem to recognize DNA mutations in tables without the prefix *c.* or with this prefix but without the dot, e.g. *c580G > T* in PMID:18549475. It also missed protein mutations, which are not point mutations, e.g. in PMID:20470368 we find *p.Glu554_Val559del*, *p.Ser566_Glu571delinsArg* and *p.Trp557_Lys558del*. In a few cases, the HGVS nomenclature used for the deletions cannot be matched directly, e.g. *c.482_483delGA* vs *c.482_483del2* or *c.501delG* vs. *c.501del1* both in PMID:15932632. Only in three cases, the gene linked to the mutation was not identified.

Looking at the false negatives from the InSiGHT database, most of the missing mutations are found in tables, either in the PMC XML file or in the article PDF. Mutations can also be found in MS Word documents, in contrast to MS Excel files for COSMIC.

There are many mentions not identified by EMU, mainly because intron and exon deletions are not expressed in HGVS format in the article tables, e.g. *Del exon 3* vs. *c.367-?_645+?del* in PMID:12373605. In some cases, there are additional formatting characters, e.g. footnote indicators as in *1704_1705del^e^AG^b^* in PMID:15655560, which are not properly handled by EMU. As for COSMIC, there are some mentions that are not identified by EMU, e.g. no prefix *c.* in *840insT* in PMID:10732761. In some cases, when curated in the database, they are converted to the most appropriate HGVS format, e.g. from *840insT* to *c.839dupT* in PMID:10732761, in which the insertion is converted into a duplication.

In one case, in PMID:17453009, a whole gene deletion was expressed in natural language (*entire gene deletion*) instead of the normalized HGVS mention (c.1-?_*+?del).Two substitutions appeared in natural language in text, e.g. *C > T mutation at nucleotide 2131* in PMID:15655560, and were missed by EMU. Finally, the position of one missed mutation mention had been corrected by the curator and thus did not match the mutation extracted by EMU, e.g. *1408-54C > T* in PMID:15655560 was curated as *c.1410-54C > T*. This position correction was indicated with a note in the database.

Even though there are only four genes catalogued by InSiGHT, there remain problems mapping the gene names, preventing some matches. For instance, *MLH1* and *MLH2* can be found as *hMLH1* and *hMLH2,* respectively, in PMID:16995940. These variants are not in *HGNC* or *EntrezGene*, even though they could be easily added to the dictionary used for gene annotation.

## Discussion

Our results clearly show that text mining of mutations from MEDLINE achieves low recall. This result alone is not entirely surprising, given previous work that showed similar effects, though in a more narrow experimental set-up (e.g. Krallinger et al. [5] focused on only two papers curated in COSMIC, including PMID:17932254 that is in our HT group with 972 mutations). We have shown that processing full-text papers is important; this is also in agreement with prior work, e.g. the analysis of protein residues in [26], as well as the general observation of differences between abstracts and full texts, with full texts argued to have more ‘content’ [33, 34]. Importantly, our results provide a novel result, quantifying the significant role that processing of additional material linked to the article with text mining plays in increasing the coverage of extracted mutations.

As shown in the false-negative error analysis in the Results section, the two databases reveal varied issues affecting matching. We find that identification of mutations other than substitutions, e.g. deletions, is problematic. In addition, the text mining tools need to be updated to cover a broader range of cases, e.g. to deal with information distributed in tabular format [32].

On the other hand, we observe some differences among the two databases we considered. There are curated mutations in the COSMIC database that are not expressed based on the gene position but rather as a chromosome offset. In InSiGHT, the main challenge is to turn explicit mentions of intron and exon deletions into HGVS format and to convert insertion and substitutions to the same normalization. Post-processing is required either to translate the identified expressions into HGVS format or to perform the calculation of the position and specific change. Furthermore, the mutations curated in the COSMIC database can be found mainly in supplementary material, while the InSiGHT mutations can be found mainly in tables.

We also found that not all mutations extracted from the corpora are curated in the databases. Mutations that appear in text may not be relevant or significant for the disease under study. For example, in PMID:10469011, the mutation *Ala140Thr* is identified by EMU, but the article explicitly states this mutation *is known to be functionally silent* and so is not included in the database. As shown in Table 1, in the COSMIC database, there are articles that are not directly associated to any mutation, while we were able to identify mutations in both the abstract and the full text using EMU in 64 common articles. These mutations were properly identified by EMU, but they were either non-coding variants, the variants had no effect on the disease mentioned in the article or the variants were out of scope for the database, e.g. non-somatic.

Finally, we note that new approaches to recognizing genetic variants in text are still being explored, including the recent *tmVar* tool [9]. Although we have not yet done a full analysis using that tool, a preliminary investigation suggests that the same basic pattern of high performance on intrinsic evaluation of mutation extraction and low recall on the extrinsic tests we have performed in our experiments also holds. On the COSMIC abstract subcorpus, tmVar extracted 3187 mutations from 2032 abstracts, which is slightly lower in absolute numbers than what was found by EMU (cf. 4267 mutations from 2486 abstracts for EMU, Table 2). Although it is possible that there will be more positive matches among these results, the overall difference between the tools in the context of our experiments is likely to have only a minor impact on recall.

## Conclusion

In this work, we have performed the first direct text mining study of the recovery of genetic variants for resources that contain explicit links to the source literature for each included mutation. Our work supports the conclusion that text mining can be an effective tool supporting curation of genetic variant information, and nothing in our analysis calls the previously established good performance of text mining tools for automated extraction of genetic variants from narrative text into question. However, we have identified processing of supplementary files as critical to achieve high recall in this endeavor. Supplementary material has not previously been considered in text mining solutions; it is clearly an important target for these tools to consider.

We have shown that when considering only the narrative content of publications, the performance of text mining tools on the task of curation of genetic variant information is very low. This effect is particularly strong for research articles based on high-throughput methods. Given the high intrinsic performance of the text mining tool, we can argue that most of the variants are not present in the narrative content of publications.

We have demonstrated that processing of supplementary material using the text mining tool results in an increase in recall from 2 to 53% for the COSMIC database, and from 8 to 46% for InSiGHT. Although our conclusion that supplementary material is a critical resource for mutation curation may be unsurprising to a biologist or biocurator, we have quantified the significant impact of ignoring this material for the task we explored.

Our analysis of false negatives suggests that the majority of the remaining missing variants can also be found in the supplementary material, but that current text mining tools, designed for processing of narrative text, are not entirely suitable for the semi-structured and varied nature of additional files. Tables and supplementary materials represent mutations in a different and more varied way, including splitting elements of a genome sequence change across different columns of structured file in diverse ways. Our results clearly indicate that a text mining system that supports curation of genetic variant data must consider not only the text but also additional material associated to published articles. However, many text mining tools, e.g. tmVar, rely on sequence classifiers that are expected to be used with data of the same type they were trained on, specifically sequences of characters or tokens of natural language text. Tables and supplementary material are not of the same type, and a sequence classifier trained on text would not be expected to work well on data with such different characteristics. Additional work is required to make such tools work robustly with both types of data.

It can be argued that the development of more robust tools to handle the complexity of supplementary materials is not the appropriate solution to the problem of recovering genetic variants from publications; that publications should instead include clearly structured data in a standard semantically specified representation (e.g. a nanopublication [35]). We support efforts towards requiring direct deposition of mutation data into central repositories that can be referred to in a publication, avoiding the need for manual curation of this data. Providing the data alongside the publication in a consistent structured format would be ideal. However, even if such changes were implemented tomorrow and there was full compliance with these standards, an extremely unlikely scenario, we will still have the problem of extracting information from the 22 million publications currently indexed in PubMed. The information locked in unstructured or semi-structured form within these publications, and the publications that will undoubtedly continue to appear with *ad hoc* supplementary material, is valuable and requires extraction. Furthermore, even if data were provided in a structured format, both the structure and the content of the data might be inadequate for future needs, therefore potentially necessitating further processing of the text in any case.

## Future Work

A large proportion of genetic variants are found in tables and supplementary material associated with the published literature. We plan to improve coverage of genetic variant extraction tools by developing targeted methods for mutation extraction in tables and supplementary material, starting with extending previous work [32]. Special retrieval strategies might be required, as full-text articles, supplementary material and tables are difficult to obtain automatically.

Our work has focused on the coverage of an automatic genetic variant extraction tool, but as mentioned before, there are mutations in the articles that are not curated in the databases. We would like to filter the delivered variants according to the specific focus of the databases, e.g. germline variants for InSiGHT and somatic variants for COSMIC, to identify those variants of direct interest to a given curation context. In addition, variant information is relevant within the context of the disease under study. We plan to extend this work, linking the extracted mutations to the diseases under research and identifying the change of function of the mutated gene product, by taking advantage of the annotated mutation-disease relationships in the Variome Corpus [11].
